# Cost-effectiveness of Intermittent vs Continuous Pulse Oximetry Monitoring in Infants Hospitalized With Stabilized Bronchiolitis

**DOI:** 10.1001/jamanetworkopen.2022.43609

**Published:** 2022-11-23

**Authors:** Myla E. Moretti, Jathishinie Jegathisawaran, Gita Wahi, Ann Bayliss, Ronik Kanani, Catherine M. Pound, Mahmoud Sakran, Patricia C. Parkin, Sanjay Mahant

**Affiliations:** 1 Clinical Trials Unit, Ontario Child Health Support Unit, The Hospital for Sick Children, Toronto, Ontario, Canada; 2 Institute of Health Policy, Management & Evaluation, University of Toronto, Toronto, Ontario, Canada; 3 Child Health Evaluative Sciences, The Hospital for Sick Children, Peter Gilgan Centre for Research and Learning, Toronto, Ontario, Canada; 4 Division of General Pediatrics, Department of Pediatrics, McMaster University and McMaster Children’s Hospital, Hamilton, Ontario, Canada; 5 Children’s Health Division, Trillium Health Partners, Mississauga, Ontario, Canada; 6 Department of Pediatrics, University of Toronto, Ontario, Canada; 7 Department of Pediatrics, North York General Hospital, Toronto, Ontario, Canada; 8 Department of Pediatrics, Children’s Hospital of Eastern Ontario, University of Ottawa, Ottawa, Ontario, Canada; 9 Department of Pediatrics, Lakeridge Health, Oshawa, and Queens University, Kingston

## Abstract

**Importance:**

Pulse oximetry is a noninvasive technology that is integral to the supportive care of hospitalized infants with bronchiolitis. A multicenter, randomized trial comparing the effectiveness of intermittent vs continuous pulse oximetry found similar length of hospital stay and safety outcomes, and greater nursing satisfaction, with intermittent monitoring.

**Objectives:**

To evaluate the cost-effectiveness of intermittent vs continuous pulse oximetry in hospitalized infants with stabilized bronchiolitis.

**Design, Setting, and Participants:**

An economic evaluation concurrent with a randomized trial in community and tertiary children’s hospitals in Ontario, Canada, was conducted using a probabilistic analysis. Patients were enrolled from November 1, 2016, to May 31, 2019. Data included infants aged 4 weeks to 24 months hospitalized with bronchiolitis, with or without supplemental oxygen, after stabilization. The cost-effectiveness analysis adopted a societal and health care system perspective and a time horizon from hospitalization to 15 days post-discharge. Patient level direct health care costs and indirect costs were included. Health resource use, costs, and clinical outcomes were obtained from trial data. Publicly available pricing resources were used to supplement costs. Ranges for sensitivity analysis were based on 95% confidence intervals of the trial data. All costs were reported in 2021 Canadian dollars.

**Interventions:**

Intermittent (every 4 hours) vs continuous pulse oximetry using an oxygen saturation target of 90% or higher.

**Main Outcomes and Measures:**

Costs and incremental costs.

**Results:**

Trial data from 229 infants (median [IQR] age, 4.0 [2.2-8.5] months; 136 boys [59.4%], 93 girls [40.6%]) were included. Mean societal costs per patient were $6879 (95% CI, $3393 to $12 317) in the intermittent and $7428 (95% CI, $1743 to $25 011) in the continuous group with a mean incremental cost of −$548 (95% CI, −$18 486 to $8105). Mean health care system costs per patient were $4195 (95% CI, $1191 to $9461) in the intermittent and $4716 (95% CI, $335 to $22 093) in the continuous group (incremental cost, −$520; 95% CI, −$18 286 to $7358). The mean effect measure of length of stay was similar between the 2 groups: 37.4 hours (95% CI, 1.0 to 137.7 hours) in the intermittent group and 38.5 hours (95% CI, 0 to 237.1 hours) in the continuous group. One-way sensitivity analyses on all variables revealed that the findings were robust and the incremental costs were not sensitive to the uncertainty within the defined ranges.

**Conclusions and Relevance:**

In this prospective economic evaluation study, we found that costs were similar for intermittent and continuous pulse oximetry considering societal and health care perspectives. Given that clinical outcomes between monitoring strategies are comparable and that other practice considerations favor intermittent monitoring, these findings provide additional information that support the use of intermittent monitoring in hospitalized infants with stabilized bronchiolitis.

## Key Points

**Question:**

Is intermittent pulse oximetry more cost-effective than continuous monitoring for children hospitalized with stabilized bronchiolitis?

**Findings:**

This economic evaluation of a randomized clinical trial found that mean societal perspective costs per patient were similar between intermittent and continuous monitoring. Costs for the health care system perspective and mean length of hospital stay were also similar between the 2 groups.

**Meaning:**

Along with other practice considerations which favor intermittent monitoring, this economic evaluation provides additional support on the use of intermittent pulse oximetry in hospitalized infants with stabilized bronchiolitis.

## Teaser

This economic evaluation of a randomized clinical trial conducted among families of infants at Canadian hospitals compares the cost-effectiveness of intermittent and continuous monitoring of pulse oximetry.

## Introduction

Pulse oximetry is integral to the supportive care of hospitalized infants with bronchiolitis.^1^ Used in conjunction with clinical assessment, pulse oximetry provides a noninvasive means for identifying hypoxia and guiding clinical management. However, there has been increasing concern that overuse of pulse oximetry in infants with stable bronchiolitis does not add value in care and may have unintended consequences.^2,3^ Guidelines recommend using pulse oximetry intermittently rather than continuously in stable, nonhypoxic hospitalized infants with bronchiolitis.^1,3^ Yet, continuous monitoring use ranges from 2% to 92% of infants hospitalized with bronchiolitis across US and Canadian hospitals.^4^ Our 2021 multicenter trial^5^ in 6 community and children’s hospitals compared intermittent (ie, every 4-hour spot checks) vs continuous pulse oximetry in hypoxic and nonhypoxic infants admitted with bronchiolitis. The trial included both in-hospital outcomes and 15-day postdischarge outcomes. The primary end point of median hospital length of stay was similar between the 2 groups, as were safety (ie, transfer to intensive care unit, revisits to hospital), medical intervention, and parent-reported outcomes (ie, anxiety and days missed from work). Nursing satisfaction was greater with intermittent monitoring.^5^

When making a practice recommendation regarding implementing or deimplementing 1 of 2 established interventions with similar clinical outcomes, decision making should also consider other factors, including costs and family or patient preferences.^6^ However, to our knowledge, no previous pediatric studies have examined the cost-effectiveness of intermittent compared with continuous pulse oximetry strategies in bronchiolitis management. We conducted a prospective economic evaluation concurrent with our multicenter randomized clinical trial measuring both societal and health care system costs. The objective of this study was to determine the cost-effectiveness of intermittent vs continuous pulse oximetry in hospitalized infants with stabilized bronchiolitis.

## Methods

### Design

A probabilistic cost-effectiveness analysis (CEA) was conducted from a societal and health care system perspectives to compare intermittent vs continuous pulse oximetry monitoring strategies for hospitalized infants with bronchiolitis. This economic evaluation was conducted according to the guidelines set out by both Canadian and US health technology assessment agencies^7,8^ and follows the Consolidated Health Economic Evaluation Reporting Standards (CHEERS 2022) statement. The patient level data was ascertained from the clinical trial.^5^ The protocol and analysis plan for the trial and economic evaluation were prespecified and published previously.^9^ All participating sites obtained research ethics board approval to conduct the trial and the economic evaluation. All patients provided written informed consent for enrollment in the trial.

### Setting and Patients

The trial was conducted in Ontario, Canada. The Ontario government provides universal health care coverage for residents and funds hospitals through global funding budgets. The trial was a pragmatic, parallel-group, randomized trial of 229 infants hospitalized at 6 hospitals (3 community and 3 children’s) in Ontario, Canada, from November 1, 2016, to May 31, 2019. Eligibility criteria have been described elsewhere in detail.^5^ In brief, patients were aged 4 weeks to 24 months, admitted with a first episode of bronchiolitis, and with a stable clinical status. As previously reported,^5^ of the 229 infants enrolled and randomized, 114 were allocated to the intermittent and 115 to the continuous pulse oximetry groups, respectively. A stable clinical status was defined by a period of 6 hours with the same or decreasing supplemental oxygen requirement (if on supplemental oxygen), the same or decreasing respiratory rate by at least 2 measurements, respiratory rate less than 70 breaths per minute, and heart rate less than 180 beats per minute. Infants were eligible for inclusion if they were on supplemental oxygen less than 40% fractional inspired oxygen or 2 L/min by nasal prongs. Infants were then randomized to receive either intermittent pulse oximetry with spot checks performed every 4 hours or continuous pulse oximetry until discharge. In the clinical trial, children were randomized after stabilization to intermittent or continuous pulse oximetry monitoring as defined above. Duration of hospital stay was defined as time from randomization to discharge in hours. Hospitals used 1 of 2 oxygen saturation targets according to their local institutional guidelines: greater than 90% when awake and asleep, or 90% when awake and 88% when asleep. All other clinical management was guided by the treating clinicians and institutional recommendations followed national Canadian Paediatric Society bronchiolitis guidelines.^10^ Patients and clinicians were not masked. No patients were lost to follow-up for the primary outcome. The main trial analyses used the intention-to-treat (ITT) principle.

### Clinical Effect Measure

The effect measure was length of hospital stay in hours. As all infants had stabilized bronchiolitis, and no long-term sequelae were expected from the illness, a time horizon from admission to 15 days postdischarge was evaluated. This allowed for the capture of any posthospitalization health care visits related to the index admission. Duration of hospital stay was defined as time from randomization to discharge in hours. Given the relatively short time-horizon, and acute nature of the illness, we did not include any measures of health-related quality of life in the participants.

### Costs Measurement and Valuation

Patient level hospital admission costs for index admissions were obtained from each hospital’s decision support system (Table 1). At discharge, and at the end of the follow-up period, direct parent interviews with structured questionnaires were used to ascertain any additional health service use and out-of-pocket expenses during and after hospitalization. This included physician visits, return to emergency department, and any hospital readmissions. Revisits were confirmed by electronic medical chart review. Physician fees not included in the institutional costs obtained from hospitals were obtained from the government of Ontario physician fee schedule.^11^ Using the human capital approach, lost productivity costs were calculated using the average daily wage in Ontario for full-time workers.^14^ The human capital approach is a method whereby a standard wage is applied to the amount of time a respondent reports their lost productivity to be, in order to estimate the losses to society as a result of their health or caregiving activities. Parents self-reported the total days of lost productivity for one or both parents, for the duration of the index hospitalization, and for the duration of the 2 weeks after discharge. Parents also reported out-of-pocket expenses paid to additional caregivers providing care for other children in the home during the infant’s hospitalization.

**Table 1.** Prices, Quantities, and Ranges Used in Cost-effectiveness Analysis

Parameter	Reference case estimate, $^a^	Source	Range used in sensitivity analysis, $	Distribution^b^
Common direct health care costs				
Visit				
Follow-up physician	90.45	Physician schedule of benefits & fees^11^	43.45-127.02	Uniform
Return to ED	279.51	OCCI^12^	1.05-3261.95	Gamma
Emergency physician in the ED	67.45	Physician schedule of benefits & fees^11^	55.90-98.35	Gamma
Physician for admission day 1	228.02	Physician schedule of benefits & fees^11^	NA	Fixed
Physician for subsequent admission days	79.50	Physician schedule of benefits & fees^11^	NA	Fixed
Readmission during follow-up (hospital costs only)	4140.25	OCCI^12^	112.01-257 147.81	Gamma
Common indirect health care costs				
Childcare or caregiving at home (hourly rate)	24.30	Statistics Canada^13^	15.00-25.79	Uniform
Lost productivity day	226.78	Statistics Canada^14^	170.82-279.32	Normal
Direct health care-intermittent monitoring				
Index hospitalization cost	3592.61	RCT	1202.37-8111.35	Gamma
Quantity of physician visits during follow-up interval, No.	1.49	RCT	1.00-6.00	Gamma
Quantity of unscheduled ED visits during follow up interval, No.	1.30	RCT	1.00-6.00	Gamma
Direct health care-continuous monitoring				
Index hospitalization cost	4051.21	RCT	1174.68-6860.29	Gamma
Quantity of physician visits during follow-up interval, No.	1.35	RCT	1.00-6.00	Gamma
Quantity of unscheduled ED visits during follow-up interval	1.30	RCT	1.00-3.00	Gamma
**Indirect costs-intermittent monitoring**				
Time babysitting reported for index hospitalization, h	49.19	RCT	8.00-123.75	Gamma
Time caregiving at home for index hospitalization, h	52.87	RCT	5.00-144.00	Gamma
Mean average other parent out-of-pocket costs	51.36	RCT	10.00-240.00	Gamma
**Indirect costs-continuous monitoring**				
Time babysitting reported for index hospitalization with continuous, h	22.22	RCT	6.00-51.00	Gamma
Time caregiving at home for index hospitalization with continuous, h	53.69	RCT	2.50-240.00	Gamma
Mean average other parent out-of-pocket costs with continuous	33.45	RCT	9.51-87.50	Gamma

Abbreviations: ED, emergency department; OCCI, Ontario case-costing initiative; RCT, randomized controlled trial.

^a^ All costs reported in 2020 Canadian dollars.

^b^ Costs with uniform distribution use minimum and maximum values for sensitivity analysis; all other costs, which are typically skewed but have a lower bound of zero, use a gamma distribution.

Both societal and health system perspectives were evaluated. For the societal perspective, total costs for the pathway of care for all participants included both direct health care costs during hospitalization and during a 15-day postdischarge period, and indirect costs resulting from parent lost productivity across the time horizon. The health system perspective excluded lost productivity and parent out-of-pocket expenses, limiting to only health care costs incurred for the 15 days of the study.

### Cost-effectiveness Analysis

A probabilistic CEA was performed with the assistance of decision tree software (TreeAge Software). The decision tree was used to compare the 2 treatment strategies, intermittent and continuous pulse oximetry, for all participants randomized in the trial. The societal perspective represented the reference case analysis, while the health system perspective was reported as a secondary analysis.

The tree began at admission with the time horizon of the analysis continued to 15 days postdischarge. Mean total costs per patient for the entire care path were assigned at a terminal node (ie, the final outcome associated with the path). Similarly, mean total length of hospital stay was assigned as the effect measure in the terminal nodes. All probability inputs to this decision tree were informed by the clinical trial data and are summarized along with the ranges used for sensitivity analysis (Table 2). For each parameter used to calculate the mean costs, a range and plausible distribution were assigned based on the variation observed in the data set or from the publicly available sources (Table 1). All costs are reported in 2021 Canadian dollars (ie, $1 CAD approximately equal to $0.75 USD). No discounting was applied to the terminal nodes owing to a relatively short time horizon, which is typical when benefits are measured less than 1 year after the intervention.^15,16,17^ A series of 1-way deterministic sensitivity analyses were performed on the quantities and costs, with each parameter varied across the defined range or 95% CI of the observed data. This was used to examine key high-cost variables which may have been associated with the incremental costs. The primary analysis was performed probabilistically, which allowed the incorporation of uncertainty in multiple variables simultaneously. During the probabilistic analysis (PA), Monte Carlo simulations were run 10 000 times with each iteration randomly drawing a value for each parameter within the specified distribution. These simulations generated point estimates and 95% CIs for the incremental costs and incremental effectiveness.

**Table 2.** Event Probabilities and Ranges Used in Cost-effectiveness Analysis Based on Trial Data^a^

Parameter	Reference case probability estimate	Range used in sensitivity analysis	Distribution^b^
**Intermittent monitoring group**			
Lost productivity for first parent during index hospitalization	1.0000	0.4270-1.0000	β
Lost productivity for second parent during index hospitalization	0.5747	0.4641-0.6801	β
Physician visit during follow-up period	0.8780	0.7871-0.9399	β
Emergency department visit during follow-up period	0.0614	0.0250-0.1224	β
Readmission during follow-up period	0.0263	0.0055-0.0750	β
Lost productivity for first parent during follow-up period	0.2308	0.1353-0.3519	β
Lost productivity second parent during follow-up period	0.2667	0.1711-0.3814	β
**Continuous monitoring group**			
Lost productivity for first parent during index hospitalization	1.0000	0.4271-1.0000	β
Lost productivity for second parent during index hospitalization	0.6316	0.5264-0.7283	β
Physician visit during follow-up period	0.9000	0.8186-0.9532	β
Emergency department visit during follow-up period	0.0870	0.0429-0.1541	β
Readmission during follow-up period	0.0348	0.0096-0.0867	β
Lost productivity for first parent during follow-up period	0.1867	0.1060-0.2933	β
Lost productivity second parent during follow-up period	0.2078	0.1237-0.3154	β

^a^ Probabilities are derived from clinical trial data.^5^

^b^ β distributions used to express uncertainty in probabilities which are bound by 0 and 1.

## Results

### Characteristics of Study Population

A total of 229 infants were randomized (median [IQR] age, 4 months [2.2-8.4 months]; 136 [59.4%] boys, 93 [40.6%] girls). There was complete follow-up of all infants for the clinical effect measure, namely length of hospital stay, as well complete cost data for the index hospitalizations. Complete 15-day postdischarge health services and economic follow-up data were available for 82 infants (71.9%) in the intermittent group and 90 infants (78.2%) in the continuous group. Demographic characteristics were not different between those with complete follow up and those missing the 15-day structured interview.

### Incremental Costs and Effects

The primary analysis revealed minimal differences in costs between the 2 monitoring strategies, with marginally lower costs in the intermittent group compared with the continuous group, with a range of incremental costs that included zero. Mean societal costs per patient were $6879 (95% CI, $3393 to $12 317) in the intermittent group and $7428 (95% CI, $1743 to $25 011) in the continuous group (Table 3). This resulted in a decrease of $548 per patient (95% CI, −$18 486 to $8105) in the intermittent group. Mean health care system costs per patient were $4195 (95% CI, $1191 to $9461) in the intermittent group and $4716 (95% CI, $335 to $22 093) in the continuous group, resulting in lower costs of $520 per patient (95% CI, −$18 286 to $7358) in the intermittent group. As reported in the clinical trial, there was no clinically important difference in median length of hospital stay (hours) between groups.^5^ For this economic evaluation, effect measure was computed probabilistically and reported as mean length of stay. The mean length of hospital stay was 37.4 hours (95% CI, 1.0 to 137.7 hours) and 38.5 hours (95% CI, 0 to 237.1 hours) for the intermittent and continuous groups respectively, an incremental difference of 1.1 hours less in the intermittent group, which is not clinically important. The reported days of lost productivity and missed activity were summarized to determine if there was any shift in burden to the parent. Total days of lost productivity reported for both parents was similar between the 2 groups (eg, mean [SD] lost productivity of parent 1 on index admission: intermittent group, 2.034 [1.083] days vs continuous group, 2.073 [1.860] days) (Table 4).

**Table 3.** Probabilistic Analysis of Intermittent vs Continuous Oxygen Monitoring Among Infants Admitted for Bronchiolitis: Societal and Health Care System Perspectives

Strategy	Perspective	Mean (95% CI)		Cost-effectiveness
		Cost per patient, CAD$^a^	Length of stay, h	
Intermittent	Societal	6879 (3393 to 12 317)	37.4 (1.0 to 137.7)	NA
	Health care system	4195 (1191 to 9461)	NA	NA
Continuous	Societal	7428 (1743 to 25 011)	38.5 (0 to 237.1)	NA
	Health care system	4716 (335 to 22 093)	NA	NA
Incremental	Societal	−548 (−18 486 to 8105)	−1.1 (−208.6 to 123.8)	Dominant (less costly, more effective)
	Health care system	−520 (−18 286 to 7358)	NA	NA

Abbreviations: CAD$, Canadian dollar; NA, not applicable.

^a^ All costs in 2021 CAD$.

**Table 4.** Summary of Parent Lost Productivity Days as Reported by Parents

Characteristic	No. of days, mean (SD)	
	Intermittent group	Continuous group
**Lost productivity, index admission**		
Parent 1	2.034 (1.083)	2.073 (1.860)
Parent 2	1.837 (1.061)	1.771 (1.563)
Total caregiver	3.871	3.844
**Lost productivity follow-up interval**		
Parent 1	3.433 (1.730)	2.571 (0.968)
Parent 2	1.500 (0.611)	2.609 (1.133)
Total caregiver	4.933	5.180

Each iteration of the probabilistic analysis is displayed as a scatter plot of incremental costs and incremental effects (Figure). The model iterations are heavily concentrated around the center of the graph, indicating uncertainty around the point estimates and that no strategy was obviously more expensive or more effective than its comparator.

**Figure.**
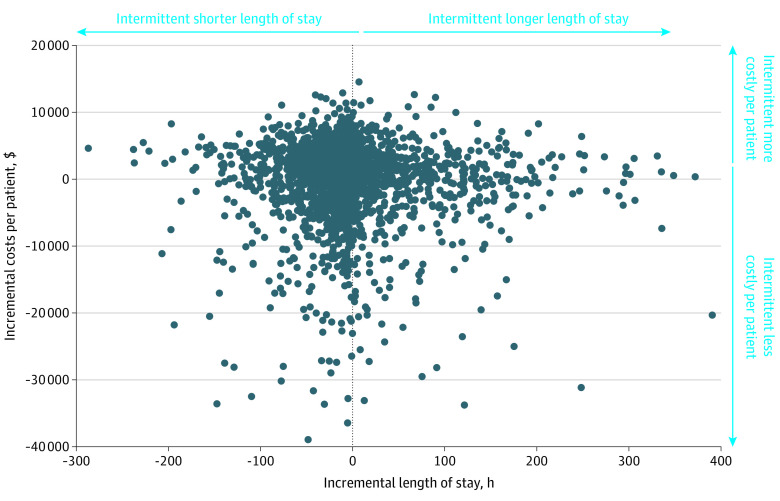
Incremental Cost vs Incremental Effectiveness Scatterplot Showing 10 000 Iterations of Probabilistic Analysis Each iteration of the probabilistic analysis is displayed as a scatter plot of incremental costs in dollars and incremental effects in length of stay in hours. The model iterations are heavily concentrated around the center of the graph, indicating that no strategy was obviously more expensive or more effective.

Although the reference case indicated the intermittent strategy was dominant, since it was both less costly and more effective (ie. it had a shorter length of hospital stay), the differences between the 2 strategies were deemed not to be clinically important, and thus it is customary not to present an incremental cost-effectiveness ratio (ICER).^17^ A reference case represents a comparison of the 2 treatment strategies when no uncertainty is accounted for. Namely, only point estimates of observed values or their means are utilized in the calculation of total case costs, effects, and incremental costs and effects.

To understand how all parameters factor into incremental costs, 1-way sensitivity analyses were performed on all variables across the ranges specified (Table 1 and 2). None of the 1-way analyses were associated with a significant change in incremental costs.

## Discussion

Bronchiolitis is the most common reason for hospitalization in children in the US and accounted for US $980 million in hospitalization costs in 2016.^18^ As hospital treatment is largely supportive, clinical and physiological monitoring is central to hospital care. Our clinical trial previously evaluated intermittent vs continuous pulse oximetry in stabilized infants hospitalized with bronchiolitis and found no clinically important difference in length of hospital stay or other clinical outcomes.^5^ In this prospective economic evaluation conducted with the trial, we found that total cost for intermittent monitoring was similar to continuous monitoring considering both overall societal and health care perspectives. Extensive sensitivity analysis further supported these findings within reasonable ranges of uncertainty. This economic evaluation used a time horizon that extended beyond hospitalization, to 15 days after discharge, and did not detect a shift in costs to the parent with the less intensive strategy (ie, intermittent monitoring). There were no meaningful differences in parent lost productivity associated with providing supportive care after discharge.

As health care systems are increasingly looking to less invasive or intensive interventions without compromising care, these findings can be used to inform bronchiolitis monitoring policies. In the case of the child hospitalized with stabilized bronchiolitis, our clinical trial and a 2015 trial^19^ indicate that clinical outcomes are comparable when oxygen monitoring is reduced to the intermittent strategy. This economic evaluation further supports this strategy, as intermittent monitoring did not appear to increase health system costs. Importantly, our study findings also show that reducing the intensity of supportive care during the hospital stay did not cause significant downstream differences in parent lost productivity days up to 15 days postdischarge and thus did not shift costs from the acute care setting to parents. Other important practice considerations also favor intermittent monitoring, such as greater nursing satisfaction, simplifying care, reducing alarm fatigue, and preparing parents for discharge home. Taken together, these findings support the guidance to use intermittent pulse oximetry in stabilized infants with bronchiolitis. A delphi expert consensus group made a recommendation to use intermittent pulse oximetry in lower severity of illness bronchiolitis in the inpatient setting.^20^

One other trial^19^ compared intermittent and continuous pulse oximetry in nonhypoxic infants hospitalized with bronchiolitis and found similar length of hospital stay and other clinical outcomes. However, that trial did not evaluate cost-effectiveness. To our knowledge, no previous studies have examined cost outcomes between intermittent and continuous pulse oximetry in bronchiolitis or other child health conditions. The use of a 90% oxygen saturation target, which was used in our trial, was found to be cost-effective compared with using a 94% oxygen saturation target in a UK multicenter trial of hospitalized infants with bronchiolitis.^21^

### Strengths and Limitations

Strengths of this economic evaluation included its parallel conduct alongside a randomized trial, with prospective data collection and prespecified cost outcomes. This evaluation used both costs and clinical effect measures from patients recruited in a pragmatic trial with real-world outcomes, rather than models based solely on estimates from the literature. In addition, half of the hospital sites were community hospitals, and almost half of the patients were recruited from community hospitals. This enhanced the generalizability of our economic evaluation as most infants with bronchiolitis are managed at community hospitals.^5^

This evaluation also had several limitations. This economic evaluation was conducted within a publicly funded health care system where hospitals receive global funding. The findings may not be generalizable to other health care systems and funding models. Although cost utility analysis is the preferred method of cost-effectiveness analysis,^8,15^ the challenges associated with obtaining utilities in children of this age as well as discerning utility changes with such a short time horizon limited our ability to perform a cost utility analysis. This evaluation was based on a trial of stabilized infants and thus the economic evaluation is applicable to the current practice standard of using continuous monitoring until stabilization. We do not have time-driven activity-based costing data for nursing care. This might have been informative to understand and explain differences in costs between interventions. For example, nursing care costs may be increased due to false alarms with continuous pulse oximetry. We did not include children with medical complexity, so our results are not generalizable to populations not included in our trial. Ambulatory health service use after discharge and lost productivity was based on direct parent interview 15 days after discharge and therefore may be subject to some recall bias. However, given the relatively short time frame it is not expected that significant health events would have been missed. Furthermore, we were able to corroborate repeat admissions when patients returned to one of the hospitals participating in the trial using medical chart review.

## Conclusions

In a prospective economic evaluation conducted alongside a clinical trial of hospitalized infants with stabilized bronchiolitis, we found that total cost for intermittent pulse oximetry was similar to continuous pulse oximetry considering both societal and health care perspectives. There did not appear to be a shift to the parent in terms of lost productivity days or out-of-pocket expenses with the less intense intermittent monitoring strategy. As clinical outcomes between monitoring strategies were comparable and other practice considerations favor intermittent monitoring, these findings provide additional information to support the recommendation to use intermittent pulse oximetry monitoring in hospitalized infants with stabilized bronchiolitis.

## Notes

Supplement.
Nonauthor Collaborators.
