# Supplementary material for: Cost-effectiveness of Intermittent vs Continuous Pulse Oximetry Monitoring in Infants Hospitalized With Stabilized Bronchiolitis
Source: JAMA Netw Open. 2022 Nov 23;5(11):e2243609. doi: 10.1001/jamanetworkopen.2022.43609 (PMC9685487; doi:10.1001/jamanetworkopen.2022.43609)
Supplement: Supplement. — Nonauthor Collaborators. [file jamanetwopen-e2243609-s001.pdf]

Moretti ME, Jegathisawaran J, Wahi G, Bayliss A, et al. Cost-effectiveness of intermittent vs continuous pulse oximetry monitoring in infants hospitalized with stabilized bronchiolitis. *JAMA Netw Open*. 2022;5(11):e2243609. doi: 10.1001/jamanetworkopen.2022.43609

Supplemental Online Content: Nonauthor Collaborators

*First name, last name, and suffix (if applicable) are required and will appear in PubMed.

***Group Name(s): Canadian Paediatric Inpatient Research Network (PIRN)**							
***First Name and Middle Initial(s)**	***Last Name**	***Suffix (eg, Jr, III)**	Academic Degrees	Institution	Location (city, state/province, country)	Role or Contribution, eg, chair, principal investigator	Group (if more than 1 Group listed in the byline) and/or Subgroup (eg, Steering Committee)
Lucy	Giglia		MD	McMaster Children's Hospital	Hamilton, Ontario	Site Member	
Anupam	Seghal		MD	Queens University	Kingston, Ontario	Site Lead	
Sean	Murray		MD	Northern Ontario School of Meidcine	Sudbury, Ontario	Site Lead	
Sepideh	Taheri		MD	Children's Hospital London Health Sciences Centre	London, Ontario	Site Lead	
Melanie	Buba		MD	Children's Hospital of Eastern Ontario	Ottawa, Ontario	Site Lead	
Peter	Gill		MD	Hospital for Sick Children	Toronto, Ontario	Vice-Chair	
